# Global Assessment of Relational Functioning: A Dynamic Family Measure Predicting Outcome in Children With Diabetes

**DOI:** 10.1111/famp.70063

**Published:** 2025-08-28

**Authors:** Marianne Côté‐Olijnyk, Peter Fonagy, Yixiao Zeng, Celia M. T. Greenwood, Alicia Schiffrin, Mona Qureshi, Zoe Atsaidis, Brian Greenfield

**Affiliations:** ^1^ Department of Psychiatry McGill University Quebec Canada; ^2^ Division of Psychology and Language Sciences University College London London UK; ^3^ PhD Program in Quantitative Life Sciences McGill University Quebec Canada; ^4^ Lady Davis Institute for Medical Research Quebec Canada; ^5^ Department of Epidemiology, Biostatistics and Occupational Health McGill University Quebec Canada; ^6^ Gerald Bronfman Department of Oncology McGill University Quebec Canada; ^7^ Department of Endocrinology Jewish General Hospital Quebec Canada; ^8^ Department of Psychiatry Jewish General Hospital Quebec Canada; ^9^ McGill University Faculty of Medicine & Health Sciences Quebec Canada; ^10^ Department of Psychiatry Montreal Children's Hospital Quebec Canada

**Keywords:** diabetes mellitus type 1, family relations, glycated hemoglobin a, parent–child relations, prognosis, treatment outcome

## Abstract

While the prevalence of type 1 diabetes (T1D) in the pediatric population has been increasing dramatically in recent years, most youths with T1D do not meet the treatment targets recommended by the American Diabetes Association. The multiple self‐report scales for parents and adolescents that have been investigated in relation to treatment adherence and glycemic control in pediatric T1D show limited predictive abilities. This longitudinal observational study investigates whether the Global Assessment of Relational Functioning (GARF) can predict the medical outcome for newly diagnosed youths with T1D. The GARF is a brief structured interview assessing important areas of family functioning. The GARF assesses three main areas of family functioning: The organization, the emotional climate, and the problem‐solving attributes of the family. Fifty‐one youths recently diagnosed with diabetes and their families were recruited from a care facility in Canada. The age of the youths ranged from 1 to 16 years (*M* = 8.89; SD = 4.2), comprising 13 preschoolers, 28 school‐aged children, and 10 teenagers. Including family members, a total of 139 people participated in the assessments. Correlations were sought between GARF scores, patients' serum glycosylated hemoglobin (HbA1c) and the frequency of ER visits, hospitalizations, episodes of ketoacidosis, severe hypoglycemia, insulin resistance, and mental health referrals over 21 months. The GARF score was significantly inversely correlated with outcome HbA1c scores (*r* = −0.61, *p* < 0.001), indicating that higher family functioning is associated with better metabolic control. These results suggest the GARF could be administered at diagnosis to predict diabetes outcome among a pediatric population.

*Healing is a matter of time, but it is sometimes also a matter of opportunity Hippocrates (c 460‐400 BCE) Precepts, ch 1*

## 1 Introduction

A diagnosis of juvenile diabetes impacts the entire family system. Youths with type 1 diabetes (T1D) and their families must adapt to a treatment regimen and share responsibilities that accompany proper management (Leonard et al. 2005). Parents describe the experience of having a child with T1D as devastating and unrelenting (Whittemore et al. 2012). The diagnosis marks the beginning of a lifelong, relentless journey filled with constant worry and time‐consuming responsibilities (Kimbell et al. 2021; Whittemore et al. 2012). Communication processes, organizational patterns, family and individual life cycles, multigenerational legacies related to illness and loss, and family belief systems will impact how a family system copes with the onset of illness in one of its members (Rolland 2018). The quality of the fit between the demands of the illness and the family's style of functioning will determine a successful adaptation (Rolland 2018). For instance, a disengaged family will have more trouble adapting to a disorder like T1D in a child, which requires regular teamwork to ensure adherence to dietary rules, specific mealtimes, regular insulin injections, and timely blood sugar monitoring (Rolland 2018). The child's illness and the family system have a mutual influence on each other. For instance, an ill child can become triangulated into pre‐existing unresolved conflicts between other family members. Children can also learn to use their condition to express their feelings and manipulate parents, especially with illnesses accompanied by life‐threatening crises such as T1D. Inversely, childhood T1D can be impacted by emotional stress and may flare up when disagreements within the parental couple escalate (Rolland 2018).

While the incidence of T1D in the pediatric population has been increasing dramatically in recent years globally (DIAMOND Project Group 2006; Gong et al. 2025; Ogle et al. 2022), the majority of youths with T1D do not meet the treatment targets recommended by the American Diabetes Association. A large study of 13,316 participants found that only 32% of youths with T1D meet age‐specific targets (Wood et al. 2013). Multiple cross‐sectional (Goldberg and Wiseman 2016; Landers et al. 2016; Pierce et al. 2017; Young et al. 2014) and prospective studies (Anderson 2004; Eilander et al. 2017) have noted an association between parenting styles and diabetes control. Factors such as high family cohesion (Cohen et al. 2004), parental warmth (Anderson 2004; Young et al. 2014), balanced boundaries, clear expectations, open communication, emotional support (Young et al. 2014), social support, and families that have already overcome challenges (Silverstein et al. 2005) are associated with better glycemic control and treatment adherence. Furthermore, just as an autonomy‐supportive style of parenting has been associated with better treatment adherence, a pathologically controlling one has been associated with defiance against diabetes self‐management in adolescents (Goethals et al. 2019).

Multiple self‐report scales for parents and adolescents have been investigated in relation to treatment adherence and glycemic control in pediatric T1D, many of which show conflicting or limited predictive abilities. A reliable measure of diabetes management often used in studies is the glycated hemoglobin (HbA1c), which reflects glycemic control over the past 3 months (American Diabetes Association 2018).

The Problem Areas in Diabetes Scale (PAID) is a self‐report scale developed to evaluate diabetes‐specific emotional distress in adults and has been adapted for use with children and adolescents and their parents (Shapiro et al. 2018). Both the teenager and parent versions of the Problem Areas in Diabetes Survey (PAID‐T and P‐PAID‐T) are associated with HbA1c levels cross‐sectionally (*r* = 0.28, *p* < 0.05 and *r* = 0.36, *p* < 0.05 respectively; Shapiro et al. 2018). The PAID‐T and P‐PAID‐T show a moderate correlation (0.44–0.48), indicating that teen and parent perceptions of diabetes‐related distress are related but not identical.

The Self‐Care Inventory (SCI) is a self‐report measure of adherence with versions validated for adolescents (ages 11–18) and parents (Lewin et al. 2009). Individuals report behaviors over the past 2 weeks on a 5‐point Likert scale. Components of the inventory include glucose monitoring, insulin administration, meal and exercise regulation, as well as keeping appointments. Parent and adolescent versions of the SCI have been found to be negatively correlated with adolescents’ HbA1c (*r* = −0.36 and *r* = −0.30, respectively, *p* < 0.001; Lewin et al. 2009).

Another measure, the self‐report Pediatric Diabetes Routine Questionnaire (PDRQ), is a parent report of the frequency of routines specific to diabetes in children and adolescents with T1D between the ages of 5 and 17. A pitfall of this measure is that no study has assessed its association with objective, clinical measures of adherence. It has been found to be moderately correlated with the SCI, another self‐report measure of adherence as described above (*r* = 0.49, *p* < 0.001; Pierce and Jordan 2012). Furthermore, a recent version developed specifically for parents of young children (PDRQ‐PYC) was found not to be significantly correlated with children's HbA1c levels (Wilcocks et al. 2022).

The Perception of Parents Scale is a self‐report questionnaire assessing the level of autonomy support provided by parents and has been adapted into a parent version and an adolescent version. The adolescents' self‐report Perception of Parents Scale score was positively associated with adolescents' HbA1c (*r* = 0.275, *p* < 0.05), but the parents' scores were not (Perlberg et al. 2021).

The scale with the most promising longitudinal predictive ability appears to be the Diabetes Family Conflict Scale (DFCS). The DFCS is a self‐report questionnaire asking parents to rate how often they argue about various tasks of diabetes management, such as recognizing symptoms of hypoglycemia, remembering clinic appointments, and telling relatives about diabetes (Hood et al. 2007). Unlike the other scales mentioned, it has been tested from diabetes onset, following youth up to 27 months into the illness, and demonstrates a small positive correlation with children's HbA1c (*r* = 0.26, *p* < 0001; Case et al. 2021).

While self‐report measures such as the PAID‐T, SCI, PDRQ, and DFCS have been useful in assessing diabetes‐related distress and self‐management, their predictive validity varies across studies. This inconsistency may result from inherent limitations of self‐report data, including social desirability and recall bias (Althubaiti 2016). In addition, discrepancies between adolescent and parent reports highlight the challenges in capturing an accurate picture of diabetes management. There is a need for further research to identify a reliable measure for predicting diabetes outcomes in this population.

The Global Assessment of Relational Functioning (GARF; Group for the Advancement of Psychiatry Committee on the Family 1996) offers a distinct approach by using observer ratings rather than self‐reports, reducing biases that can influence patient‐reported outcomes. We identified only one observer‐rated interview investigated in the context of T1D management in youth. The Diabetes Self‐Management Profile (DSMP) is a semistructured interview assessing diabetes self‐management across the preceding 3 months (Harris et al. 2000). It includes 23 questions and covers five domains, including exercise, management of hypoglycemia, diet, glycemia measurements, and insulin administration. The total score was significantly correlated with HbA1c (*r* = −0.20, *p* < 0.01) cross‐sectionally (Harris et al. 2000); no study has assessed this association longitudinally. While the DSMP is also an observer‐rated measure, it primarily assesses individual self‐care behaviors, such as insulin administration and diet adherence (Harris et al. 2000). In contrast, the GARF, by providing a clinician‐rated assessment of overall family functioning, may offer a more accurate reflection of the family's impact on disease management. Previous research has demonstrated that family conflict correlates with glycemic control (Case et al. 2021); yet, no existing study has examined the correlation between HbA1c and a structured, observer‐rated measure of family dynamics. This study aims to determine whether the GARF serves as a reliable predictor of diabetes management from onset.

The GARF, used in psychiatric settings, shows promise as a tool for investigating functioning among families of youths with T1D in a nonpsychiatric medical milieu. It consists of a brief structured interview assessing three important areas of family functioning (Mello et al. 2007; Rosen et al. 1997; Ross and Doherty 2001; Sperry 2012; Stiefel et al. 2003; Wilkins and White 2001) relevant to diabetes care: The ways the family accomplishes tasks while differentiating, maintaining, and shifting roles when necessary; the emotional climate of the family through verbal and non‐verbal communication; and the problem‐solving attributes of the family when faced with conflict. It is meant to be easily accessible to different professionals, including family physicians and researchers.

The current study aims to examine whether this measure of family functioning at diagnosis can predict long‐term adjustment to T1D. In addition, the present study was designed to test the GARF's internal consistency and interrater reliability in a pediatric context. We hypothesized that good family functioning at treatment onset would correlate with good biological outcomes at approximately 1 year follow‐up while controlling for HbA1c immediately postdiagnosis. Relatively long‐term prediction is important, given the frequent honeymoon period of good diabetic control following diagnosis, probably associated with residual pancreatic function and often lasting several months, potentially obscuring the relationship between diabetic control and family function (Abdul‐Rasoul et al. 2006). We further expected GARF scores to correlate negatively with other medical indicators of adjustment, including frequency of patients' ER visits, hospitalizations, ketoacidotic episodes, severe hypoglycemia, and a need for mental health referrals—all reflecting poor adaptation to, and control of, diabetes (Rewers et al. 2002). The pragmatic design of the study was adopted to test if GARF scores could enable clinicians working with youths with diabetes to readily identify those youths and families most at risk of a poor outcome, triggering early interventions and ensuring healthier trajectories.

## 2 Method

### 2.1 Participants, Recruitment, and Diagnosis

Participating families were consecutively recruited from an outpatient diabetes clinic associated with a large urban pediatric tertiary‐care hospital in Canada. The hospital is a teaching and research facility treating newborns, children, and adolescents up to age 18, and its volume is approximately 120, 000 visits per year in its outpatient clinics. The outpatient pediatric diabetes clinic is located within the hospital and is composed of pediatric endocrinologists and nurses, nutritionists, and social workers specializing in diabetes care. Consultants from other departments, including mental health professionals, are available upon consultation.

Ethics approval was obtained from the Research Ethics Board of the Montreal Children's Hospital (PED‐96‐1284). Written consent was obtained from participating families. All families were approached who met the following inclusion criteria: (a) they had a child between the ages of 1 and 17 years diagnosed with T1D; (b) they lived with, or near to, the identified patient; (c) they had a reasonable command of English and/or French; and (d) they were willing and able to give written informed consent for study participation. We included patients between the ages of 1 and 17 to examine the association between GARF scores and diabetes management across childhood and adolescence.

The sample consisted of 51 families of children recently diagnosed with diabetes. Only two families meeting these criteria, of the 53 approached, refused to participate in the study. All families agreed to participate in direct interactional assessments within 19 weeks of diagnosis. All data were collected before any psychosocial interventions were initiated, and the study was approved by the hospital ethics committee. Fifty‐one interviews were conducted. Although physically present, children younger than 6 years of age (*n* = 13) did not participate in the interview. Fathers who could not be present were replaced by another family member, except in one case. A total of 139 people participated in the assessments.

The age of the participants ranged from 1 to 16 years (*M*_*age*_ = 8.89; SD = 4.2). Participants included 27 males (53%). Thirty families (59%) spoke English as their mother tongue. The sample comprised 13 preschoolers, 28 school‐aged children, and 10 teenagers. Fifty‐nine percent of the interviews were conducted in English and 41% in French. Nine of the families spoke neither English nor French at home. Ninety‐eight of the interviewees were White Canadians, seven were Black Canadians, four were Latin American Canadians, and four were East Indian Canadians. No child in the sample had an additional chronic illness, and none had previously consulted a psychiatrist; although one family was scheduled to do so for a child with “behavioral problems.”

### 2.2 Training of Raters

The two raters consisted of a nurse with a master's degree in nursing and a child psychiatrist, each with at least 13 years of clinical experience working with families. After studying the GARF scales and manual and learning the operational definitions of family organization, problem‐solving, and emotional climate, they scored videotaped vignettes exemplifying the three categories of GARF functioning. In each vignette, core constructs were discussed and operational definitions reconsidered. Once the raters consistently achieved scores within 10 points of each other (on the GARF 100‐point scale) on ratings of five consecutive families, testing of the families of patients with diabetes began.

### 2.3 Design

Informed consent was obtained from 51 youths and their families presenting consecutively to the hospital's diabetes clinic. After the three standard and routine postdiagnosis information sessions, the GARF was administered by the raters to determine the measure's reliability and establish a baseline measure of a family's capacity to communicate, demonstrate role flexibility, and solve problems, all of which would be reflected in their relationship with the treatment team. The six questions administered in the same sequence to each family can be found in the Appendix.

These patients were followed during the next 21 months of routine diabetic care, while regularly recording their serum HbA1c levels. Regular recordings were made of the frequency of patient ER visits, hospitalizations, episodes of ketoacidosis, severe hypoglycemia, insulin resistance, and mental health referrals (i.e., social worker, psychologist, psychiatrist). These were recorded at patients' follow‐up appointments at the diabetes clinic and identified by the research team through chart review. Patient levels of HbA1c were tested monthly. Owing to variability as to when patients exit the honeymoon period, notation was made of the first time the patient's HbA1c levels exceeded 48 mmol/mol (6.5%) postdiagnosis.

## 3 Instruments

### 3.1 The Global Assessment of Relational Functioning

This is a brief structured interview assessing important areas of family functioning (Mello et al. 2007; Rosen et al. 1997; Ross and Doherty 2001; Sperry 2012; Stiefel et al. 2003; Wilkins and White 2001). It provides valuable information about the family context, is inexpensive, and can be administered easily and rapidly to families in clinical settings (Dausch et al. 1996). It reveals how families adapt to the stresses associated with a newly diagnosed chronic illness and does not require extensive training for administration. The GARF has three key facets. First, structural characteristics describe how families accomplish tasks, including differentiating and maintaining roles when responding to individual needs and shared expectations, shifting roles when necessary, and modifying lines of authority, responsibility, and power. Second, the emotional climate assesses the quality and range of emotions expressed through verbal or nonverbal communication and the concern experienced between family members. Third, problem‐solving attributes gauge the family's ability to identify and resolve conflict, negotiate shared plans for daily activities, and communicate clearly.

When the GARF was administered in psychiatric settings to families of patients with bipolar disorder, the intraclass correlation coefficients (ICC) obtained between the criterion rater and the trained raters (0.81–0.94) and among the trained raters (0.72) were good (Dausch et al. 1996). However, reports vary, with interrater reliability being reported as modest (Rosen et al. 1997; Wilkins and White 2001), high (Denton et al. 2010; Hilsenroth et al. 2000; Stein et al. 2009), and very high for raters trained in systemic family approaches (Mottarella et al. 2001). The GARF has a high internal consistency (Stein et al. 2009) and good concurrent validity (Denton et al. 2010). Furthermore, good convergent validity was observed between the GARF and the Social Cognition and Object of Relations Scale (SCORS), suggesting that the GARF is a reliable measure of relational functioning (Stein et al. 2009). For the purposes of our study, a GARF score of 60 was empirically determined as the cutoff to define good family functioning, based on the scale provided by the authors of the GARF (Group for the Advancement of Psychiatry Committee on the Family 1996) as well as on the clinical experience of the authors. According to the five 20‐point categories that comprise the GARF, a cutoff of 60 differentiates families that are “functioning satisfactorily” and “somewhat unsatisfactory” from those classified as “clearly dysfunctional,” “obviously and seriously dysfunctional”, and “too dysfunctional to retain continuity of contact and attachment” (Group for the Advancement of Psychiatry Committee on the Family 1996).

### 3.2 Glycosylated Hemoglobin A1c

There is a direct relationship between mean glucose concentration and HbA1c values over the red blood cell's life, spanning 120 days on average (Wilson et al. 2011). A high HbA1c corresponds to poor diabetes control in the months before. Furthermore, there is strong evidence that improved glycemic control decreases the onset and progression of long‐term complications related to diabetes (Nathan et al. 1993; Wherrett et al. 2013).

## 4 Statistical Analysis

Pearson correlations and paired sample *t*‐tests were used to compare the two raters' scores on the GARF. Scale reliability was assessed with Cronbach's alpha and Pearson correlations. Since biological outcomes were measured dichotomously, chi‐square tests were used to assess the relationship between biological outcomes and GARF scores with a cutoff point of 60. Pearson correlations and partial correlations were examined between the GARF overall scores and patients' follow‐up HbA1c scores with HbA1c at onset as covariate. The software SPSS version 24 was used for the analysis of data. Concerning power, a sample size of 51 is sufficient to detect a medium (*r* = 0.3) to large (*r* = 0.5) effect with Pearson correlation or chi‐square contingency tests using an α = 0.05 and β = 0.2 (Cohen 1992).

Multiple imputation‐based sensitivity analysis was performed to test the robustness of the Pearson correlation and partial correlation results to the patterns of missingness in HbA1c. In order to ensure the reliability of the imputation, 11 patients with more than 50% missing measurements in follow‐up HbA1c were not included for imputation, leaving 40 patients, including 30 in the high GARF group and 10 in the other. The missing rate of HbA1c ranged from 0% to 44.4% for the remaining patients. The multiple imputation of HbA1c was implemented by R package MICE (version 3.13.0; Buuren and Groothuis‐Oudshoorn 2011). The imputation method was set to predictive mean matching, and age, clinical exposures (reference to mental health services, poor control, episodes of severe hypoglycemia, insulin resistance, calls to clinic), together with measured HbA1c, were used as the predictors in imputation models. Final estimates and statistical inference were pooled from the analyses on multiple imputed datasets (Little and Rubin 2020).

## 5 Results

This study demonstrates that better family functioning, as assessed by the GARF, is associated with lower HbA1c levels, indicating better metabolic control. A strong negative correlation (*r* = −0.61, *p* < 0.001) was found between the posthoneymoon score of HbA1c and the GARF total score at diagnosis (*N* = 29). The correlations between the GARF total score and HbA1c between 12 months (*N* = 44) and 21 months (*N* = 31) were all significant (*r* = −0.53, all *p* < 0.001) while correlations between the GARF score and HbA1c prior to 12 months were all nonsignificant (*p* > 0.1). These significant correlations were maintained with partial correlation, controlling for HbA1c at diagnosis. Mean HbA1c for participants who scored above and below the cutoff of 60 can be found in Table S1. These inverse relationships between GARF scores and posthoneymoon HbA1c levels remained very similar after imputation of missing data. As shown in Figure S1, the point estimates of pairwise Pearson correlation from the unimputed dataset were close to the medians or within the interquartile ranges of the distributions generated from 200 bootstrapped datasets followed by imputations. A similar plot for the partial correlations controlling for HbA1c at diagnosis is provided in Figure S2.

The results of correlations and partial correlations before and after imputation, paired with the corresponding number of samples, are summarized in Table S2. Of note, the inferences for partial correlations demonstrated dramatic improvements from the multiple imputation process when comparing the results estimated on complete data only, given how few observations contributed to this analysis prior to imputation. Although multiple imputation of missing measurements introduces some additional uncertainty in correlation inference, the conclusion that GARF scores are negatively correlated with long‐term HbA1c prediction is robust to the missing data patterns. Results of diagnostic tools for inspecting the imputation quality, including (a) comparing the distribution of imputed values to that of observed values and (b) examining the prediction performance of the imputation models on observed data, are provided in Figures S3–S6.

GARF scores (*M* = 73.96, SD = 13.63) were approximately normal in distribution according to P–P plots, with skew = −0.73. Thirteen patients scored at or below 60 and 38 above. Concerning the second study aim, high inter‐rater reliability was achieved between the two raters on the total GARF scores (*r*(26) = 0.95, *p* < 0.001), and no significant mean differences were observed, *t*(26) = 0.825, *p* = 0.42. Tests of internal consistency yielded a Cronbach's alpha of 0.97 for the three GARF subscales, and inter‐subscale correlations were very high (*r*_51_ = 0.90–93). Problem‐solving correlated highly with organization (*r* = 0.899) and emotional climate (*r* = 0.932), and emotional climate almost perfectly predicted family organization (*r* = 0.921).

There was no significant association between our primary outcome, family function, and the age of the participants (*r*_51_ = 0.12, *p* = 0.4). With respect to the expected association of family function with clinical variables, a GARF score of 60 was used as a cutoff point for poor or good family function. No significant relationships (*p* > 0.3) were found in chi‐square tests between the patients' scores on either the GARF and the presence or absence of episodes of severe hypoglycemia, indications of insulin resistance, and referral to mental health. Point bi‐serial correlations between the GARF score and these clinical variables were nonsignificant (Table S3). Additionally, no patient experienced an episode of diabetic ketoacidosis, and none were hospitalized.

## 6 Discussion

The key finding of the current study was the capacity of the GARF to anticipate HbA1c levels at 12 months and beyond in a sample of families of children and adolescents. The wide age range included suggests that the GARF shows promise in identifying a broad age range of youths at risk of a poor outcome. The correlation observed was large and statistically significant, indicating that 35% of the variance in glycosylated hemoglobin could be predicted from a 20‐min standardized observation of family functioning. In fact, the predictive relationship remained significant for 21 months following diagnosis. This quite promising result suggests that at‐risk youths with newly diagnosed T1D could be identified early on the basis of their family functioning. Early identification of these youths and their families could be used to promote early psycho‐social intervention, including efforts to improve family functioning, to proactively improve their prognosis (Silverstein et al. 2005).

The instrument used to assess family function, the GARF, performed well, and a high inter‐rater reliability was achieved, comparable to other studies using this instrument (Denton et al. 2010; Hilsenroth et al. 2000; Stein et al. 2009) but this time administered to families of youths with new‐onset diabetes. Interviews were performed in the pediatric setting as per routine diabetic education, suggesting that the GARF could be routinely applied in clinical settings where youths with T1D are diagnosed and treated. We observed very high associations between subscales of the GARF, in line with previous studies using this instrument (Stein et al. 2009). The high coefficient of consistency observed raises the possibility of a simpler, perhaps even shorter, assessment using just one or two subscales in future clinical applications, which should be explored in future studies.

We hypothesized that the GARF scores would be related to other clinical indicators of adaptation to the diagnosis, such as the frequency of visits to the ER, hospitalizations, episodes of ketoacidosis, severe hypoglycemia, and a need for referrals to mental health resources (e.g., social worker, psychologist, psychiatrist). No relationships were found between GARF scores and these outcomes. It is possible that these indicators are more likely to emerge as indices of adjustment later in the course of the disease as they are likely the consequence of poor adherence to a diabetic regimen. The absence of significant relationships may also relate to the binary coding of these events and the subsequent use of a less‐sensitive statistical test.

Our results are in line with previous studies on family dynamics and T1D in children and adolescents (Case et al. 2021). The GARF demonstrates a stronger correlation compared to the DCFS and the DSMP in assessing T1D management in youth, despite encompassing a more diverse age range in our sample.

The GARF distinguishes itself from other promising measures, such as the DCSF and the DSMP, by its focus on family dynamics rather than behavioral compliance, in combination with being an observer‐rated measure. Furthermore, its predictive ability is strengthened by the persistence of its association with diabetes outcomes up to 21 months into the illness.

## 7 Limitations

A limitation of our study is that the present initial investigation is based on a small sample, with only two raters and a population of youths with a wide age range. Also, they are drawn from a single clinic, which may not be representative of the clinical population. It would be useful to replicate this investigation in a multi‐site setting to further extend the generalizability of this study and to validate the GARF's application with trained staff without a background in mental health or family therapy. In addition, since the study was conducted, new predictors of good glycemic control have been developed, including target range to assess glycemic variability. Future studies should include this measure. This study controlled for HbA1c at onset, but did not control for other potential confounders, including socioeconomic status of the family, which can affect the trajectory. There was also significant attrition in the sample size over the course of the study that may have influenced relationships between variables at the later time points. A number of patients (and their families) were lost to follow‐up. There were 29 patients at baseline (59.6% completion rate), reaching a maximum of 51 patients at the 3‐month follow‐up (91.1%) and then slowly trickling to 44 patients at the 12‐month follow‐up (86.2%) and 31 patients at the 21‐month follow‐up (60.8%). There is a risk of bias considering patients lost to follow‐up may have had a different outcome than their GARF assessment predicted. Hence, we undertook a sensitivity analysis to examine the robustness of these results to the missingness. Results after multiple data imputations showed remarkable consistency with the original findings, so that we can conclude these findings are unlikely to be the result of biased missing data patterns. Furthermore, we included all patients consecutively presenting to a diabetes clinic in a tertiary care teaching hospital, and therefore a large age range, from 1 to 16 years old. It would be interesting for further studies to test the GARF's predictive abilities on a narrower age range.

## 8 Clinical Implications

Findings from this study suggest that the GARF could be a valuable tool in pediatric diabetes clinics to assess family functioning at diagnosis and identify families who are at higher risk of poor outcomes. Early identification of these families could facilitate timely interventions to better support them and attempt to alter the trajectory of illness. For instance, families with a low GARF score (e.g., ≤ 60) could be referred for evidence‐based family interventions.

The GARF is designed to be used by a wide range of professionals, including mental health professionals, family physicians, and researchers, when assessing a child with a new diagnosis of diabetes. It comes with an instruction manual and case vignettes, and training is relatively time efficient. Most clinicians become proficient after 1.5 to 2 h of training, with those experienced in family interviewing requiring only about half that time (Group for the Advancement of Psychiatry, 1996). Beyond hospital‐based diabetes care, behavioral health professionals working in community settings could also use the GARF to identify children with newly diagnosed T1D at risk of poor outcomes and provide structured support around communication, problem solving, and role distribution. Given the limited availability of family‐focused psychosocial services, this approach may help prioritize access to these precious but often limited resources for families who need them the most.

## 9 Conclusion

Our goal was to adapt an existing family measure of psychological functioning to a standard initial assessment of youths presenting with insulin‐dependent diabetes, accompanied by their parents/other family members. We hoped that this measure would require little training for application in most clinical settings and correspond with treatment outcome. We demonstrated that an easily administered and quantifiable measure, such as the GARF, which could be quickly integrated into a routine assessment of pediatric diabetes at the outset of treatment, can reliably alert the clinician to a potentially poor treatment response by 12 months following the initial diagnosis of diabetes. This simple tool would allow for better long‐term treatment planning and could result in better outcomes for the patients. Despite these findings, many questions remain unanswered. Future research should explore how these associations continue to evolve after 21 months of follow‐up, as T1D is a lifelong illness. It would also be interesting to replicate the study with larger samples, considering quality of life and potential mediators of the associations between family dynamics and HbA1c (e.g., social determinants of health, diet, and exercise, to name a few).

## Conflicts of Interest

The authors declare no conflicts of interest.

## Supporting information


**Appendix S1** Clinical interview administered to families.

**Appendix S2** Supporting Information.

## Data Availability

The data that support the findings of this study are available from the corresponding author upon reasonable request.
