# Supplementary material for: Global Assessment of Relational Functioning: A Dynamic Family Measure Predicting Outcome in Children With Diabetes
Source: Fam Process. 2025 Aug 28;64(3):e70063. doi: 10.1111/famp.70063 (PMC12394924; doi:10.1111/famp.70063)
Supplement: Supplementary file 1 — Appendix S1 Clinical interview administered to families. [file FAMP-64-0-s001.docx]

**Appendix 1**

The following six questions were administered in the same sequence to each family:

1. Can you please tell us how you felt and reacted when you were told that your child was diagnosed with diabetes?
2. What was the most difficult part of finding out about the diabetes, and how did you deal with this?
3. Which family member was most affected by the diagnosis?
4. Some couples say that difficult experiences in life, like having a child diagnosed with diabetes, bring the couple closer together, while other couples maintain that such experiences have created distance in the couple. Which of these two conditions best characterizes how this diagnosis has affected your couple relationship?
5. What advice and strategies would you give and recommend to a family approaching you with the following hypothetical problem?
   1. (For families whose child is a toddler) the child refuses to eat, or runs away from the parent at the time of the injection; or,
   2. (For families whose child is aged three to 17 years) the child with newly diagnosed DM is caught consuming sweets without telling his parents (“cheating”).
6. Could you describe a challenging situation you previously faced together as a family, and how you dealt with it?
