# Supplementary material for: Global Assessment of Relational Functioning: A Dynamic Family Measure Predicting Outcome in Children With Diabetes
Source: Fam Process. 2025 Aug 28;64(3):e70063. doi: 10.1111/famp.70063 (PMC12394924; doi:10.1111/famp.70063)
Supplement: Supplementary file 2 — Appendix S2 Supporting Information. [file FAMP-64-0-s002.doc]

**Supplemental Materials**

**Figure S1**

*Boxplots of Pairwise Pearson Correlations Between GARF Scores and HbA1c Levels at Each Point in Time*


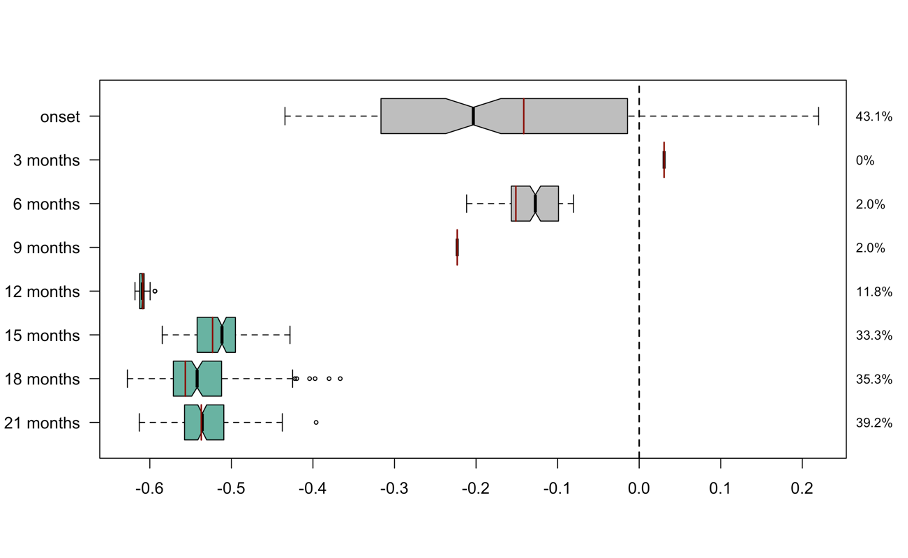


*Note.* The boxplots were generated from analyses of 200 sets of imputed data. The red vertical lines indicate the pairwise correlations on the original data without any imputation, while the y axis on the right side describes the percentage of samples lost when calculating such correlations without imputation.

**Figure S2**

*Boxplots of Partial Correlations Between GARF Scores and HbA1c Levels at Each Point in Time*
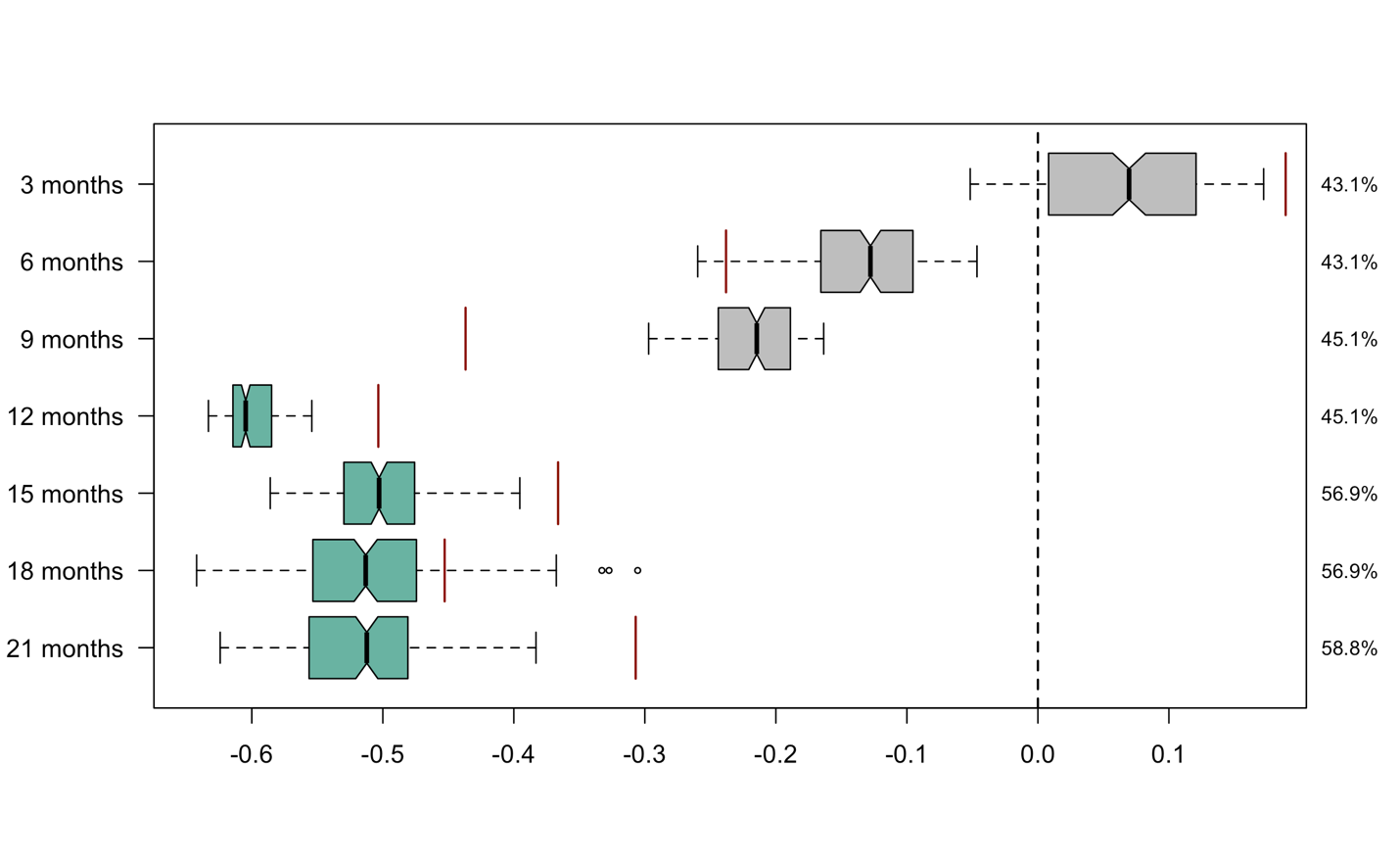


*Note*. The boxplots were generated from analyses of 200 sets of imputed data. The red vertical lines indicate the complete-case partial correlations on the original data without any imputation, while the y axis on the right side describes the percentage of samples lost when calculating such partial correlations without imputation.

**Figure S3**

*The Distribution of Imputed Missing Values and Observed Values for HbA1c Measured from 15 Months to 21 Months*


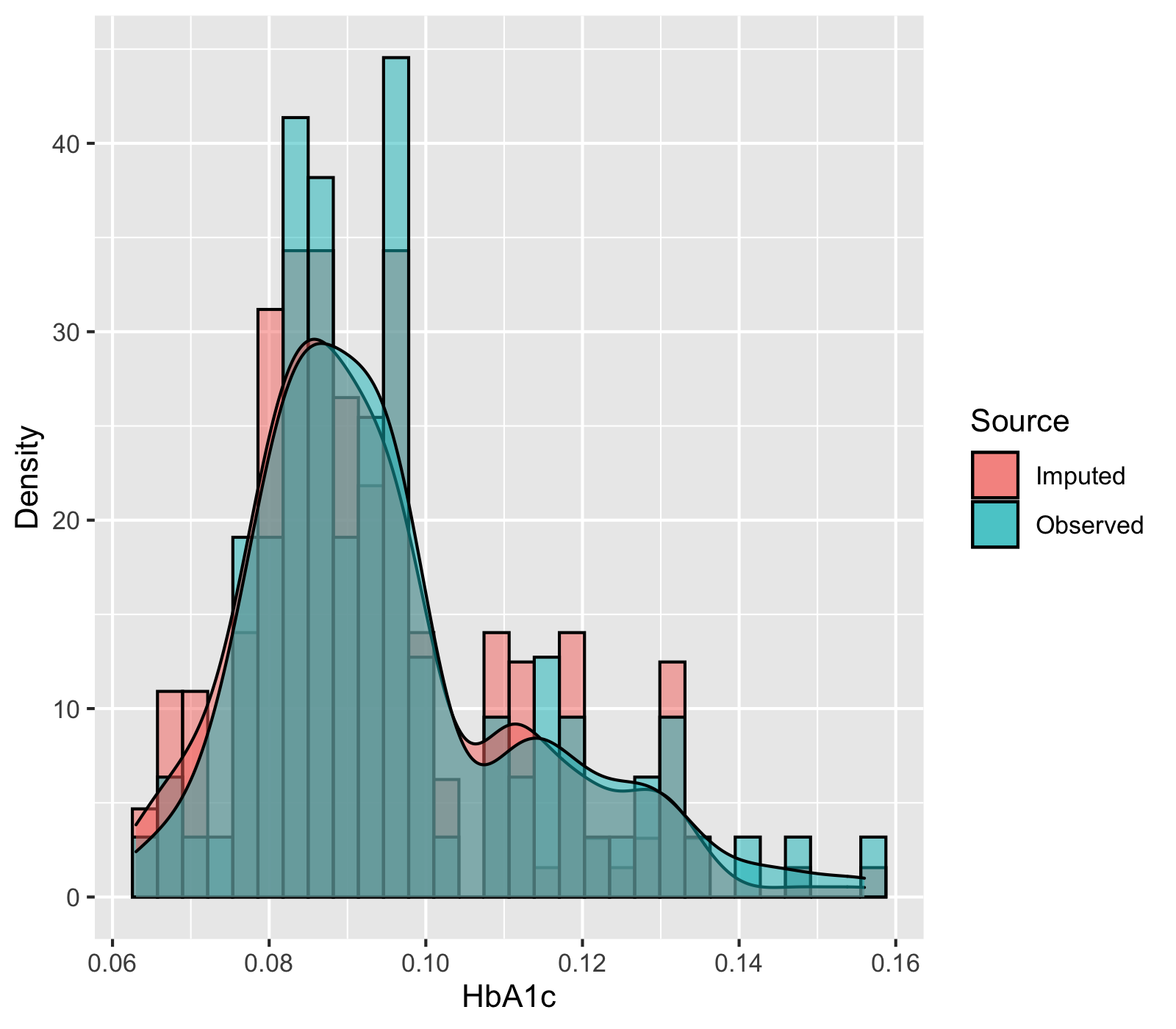


*Note*. Imputation with a strange distribution distinct from the observed one may indicate that the imputation model needs some improvements. The plot was generated by 200 multiple imputations.

**Figure S4**

*The Distribution of Imputed Missing Values and Observed Values for HbA1c Measured at Diagnosis*


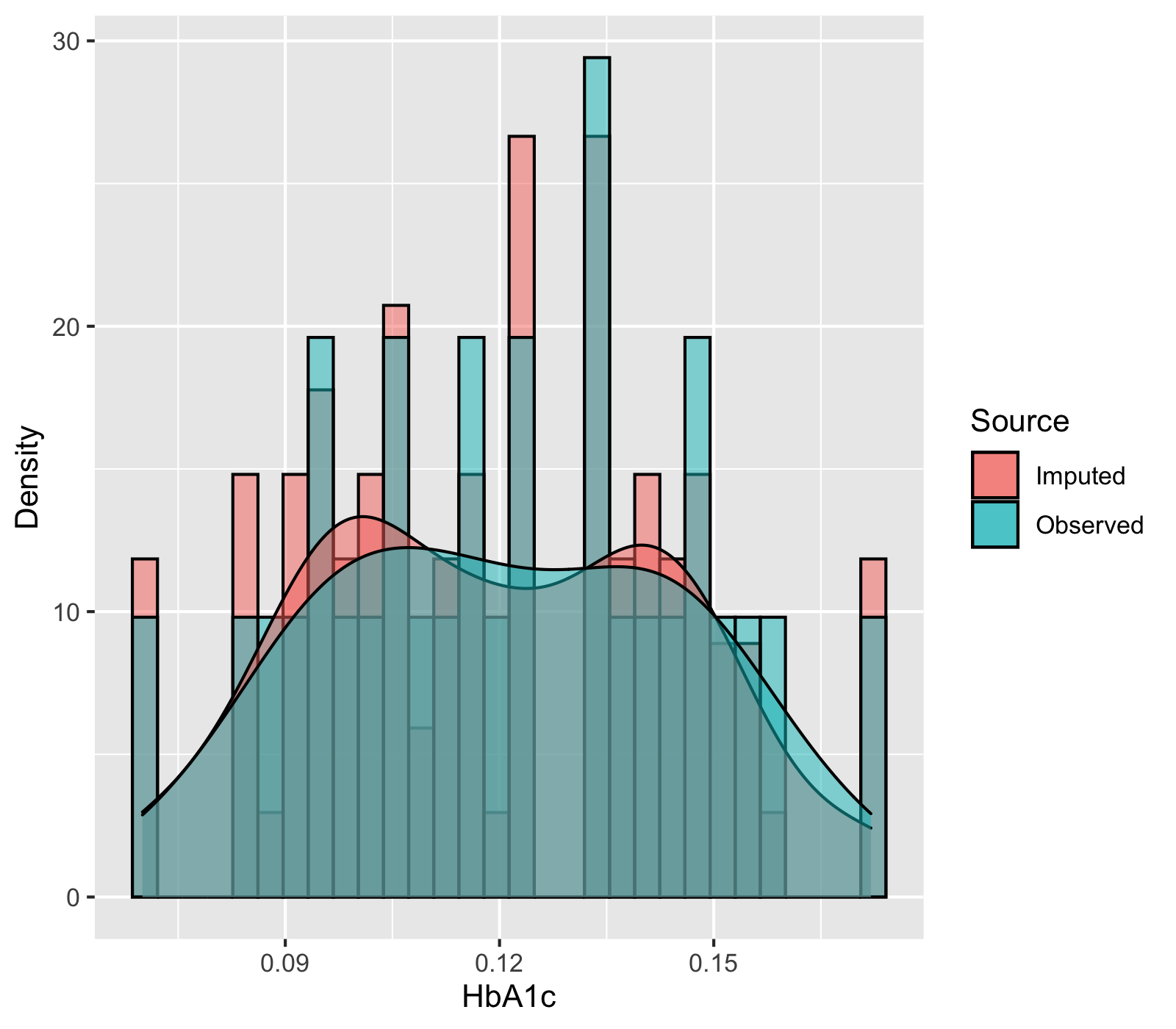


*Note*. The plot was generated by 200 multiple imputations.

**Figure S5**

*Over-imputed Plot for the Observed HbA1c Values Measured from 15 Months to 21 Months*


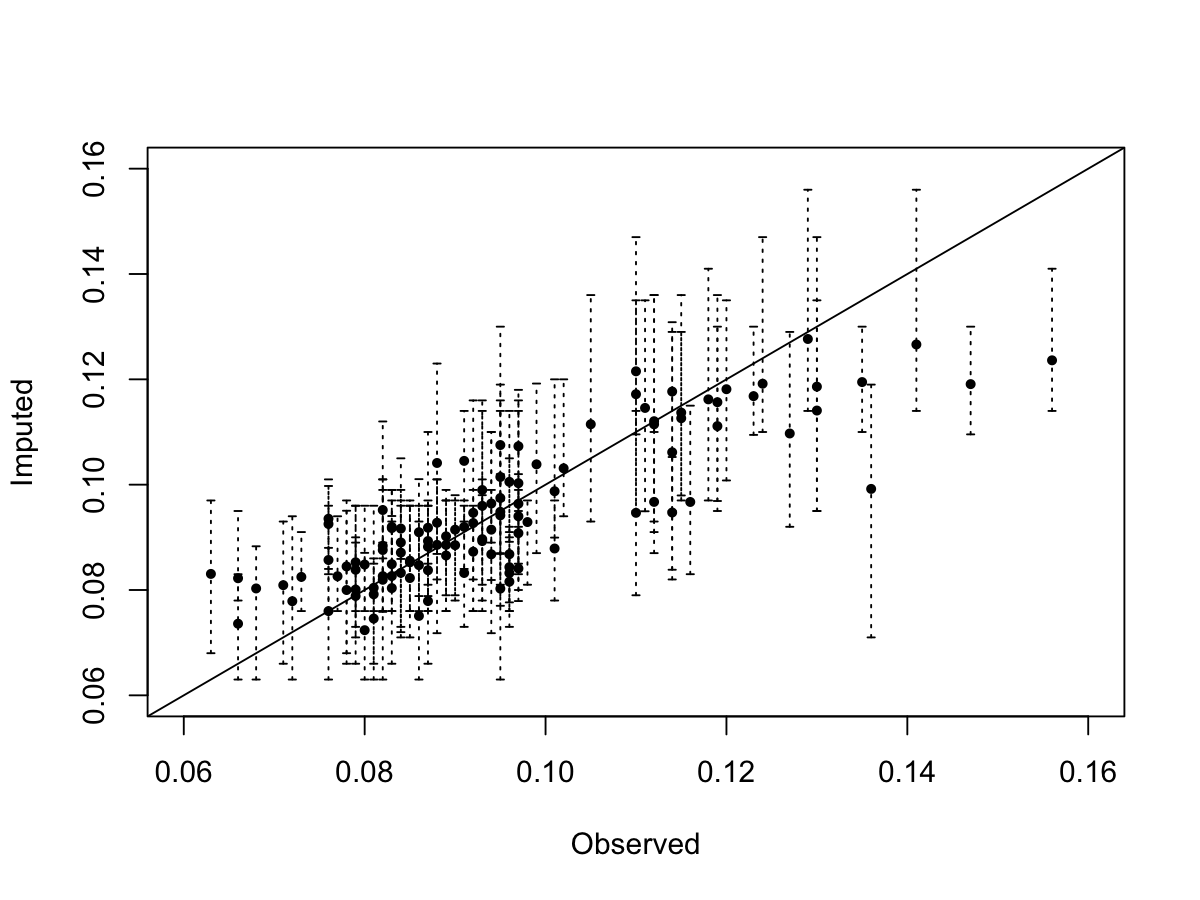


*Note*. Points and dashed lines represent the means and 95% confidence intervals of over-imputed observed values. Over-imputing sequentially treats each of the observed values as if they had been missing and then co-imputes this value with existing missing values. The y = x line indicates the line of perfect agreement between observation and imputation. The plot was generated by 200 multiple imputations for each of the observed values.

**Figure S6**

*Over-imputed Plot for the Observed HbA1c Values at the Point of Diagnosis*


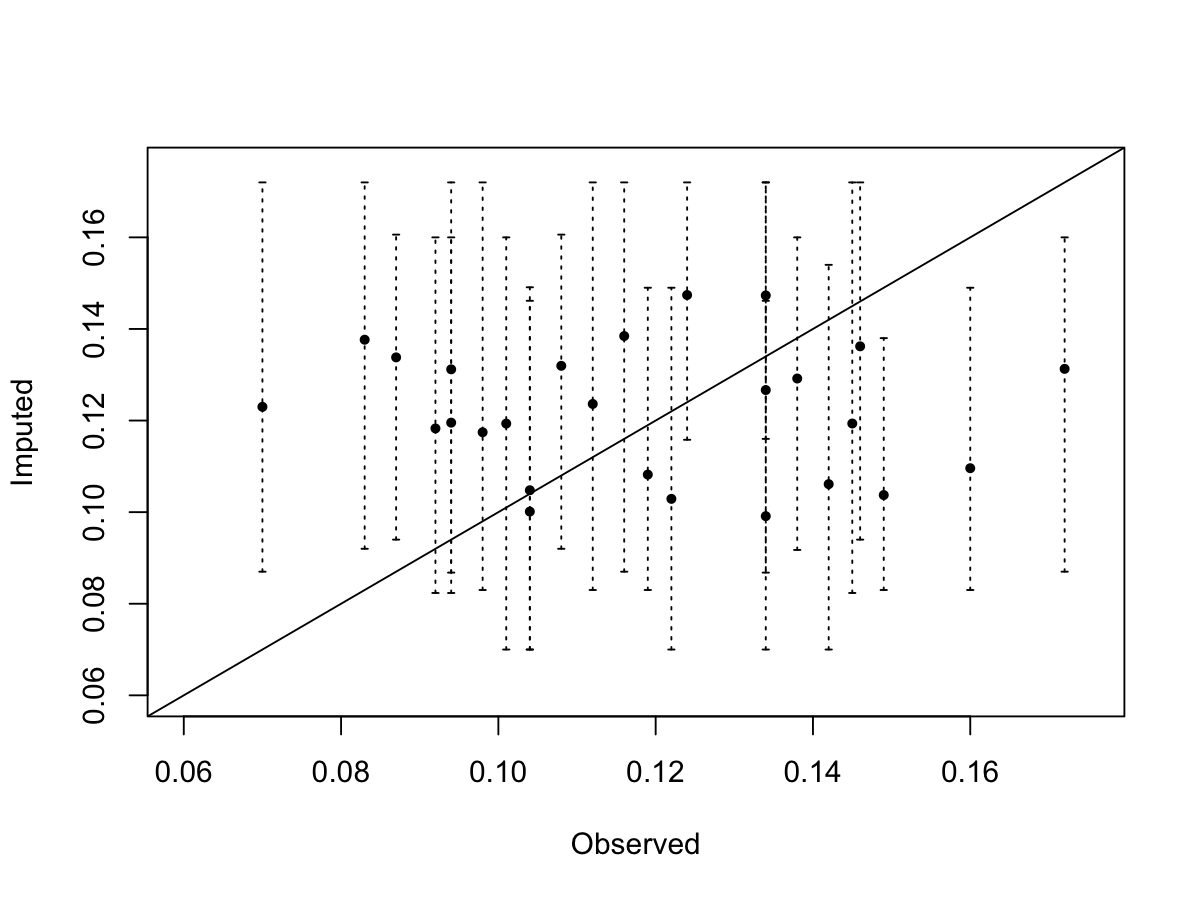


*Note*. Points and dashed lines represent the means and 95% confidence intervals of over-imputed observed values. Over-imputing sequentially treats each of the observed values as if they had been missing and then co-imputes this value with existing missing values. The y = x line indicates the line of perfect agreement between observation and imputation. The plot was generated by 200 multiple imputations for each of the observed values.

**Table S1**

*Mean HbA_1c_ for Participants Scoring At or Below and Above 60 on* the GARF

| GARF average | N | Mean HbA1c, % | Mean HbA1c, mmol/mol | Standard Deviation | Standard Error Mean |
| --- | --- | --- | --- | --- | --- |
| At onset |  |  |  |  |  |
| ≥ 61.0 | 23 | 11,843 | 105 | .024303 | .005067 |
| < 61.0 | 6 | 12,933 | 117 | .031860 | .013007 |
| At 3 months |  |  |  |  |  |
| ≥ 61.0 | 38 | 9,145 | 76 | .014682 | .002382 |
| < 61.0 | 13 | 8,900 | 74 | .013298 | .003688 |
| At 6 months |  |  |  |  |  |
| ≥ 61.0 | 38 | 8,668 | 71 | .012968 | .002104 |
| < 61.0 | 12 | 9,383 | 79 | .019021 | .005491 |
| At 9 months |  |  |  |  |  |
| ≥ 61.0 | 37 | 9,000 | 75 | .010143 | .001668 |
| < 61.0 | 13 | 10, 038 | 86 | .022995 | .006378 |
| At 12 months |  |  |  |  |  |
| ≥ 61.0 | 33 | 8,885 | 74 | .010628 | .001850 |
| < 61.0 | 11 | 11,027 | 97 | .019453 | .005865 |
| At 15 months |  |  |  |  |  |
| ≥ 61.0 | 27 | 9,104 | 76 | .012744 | .002453 |
| < 61.0 | 7 | 11,829 | 105 | .026203 | .009904 |
| At 18 months |  |  |  |  |  |
| ≥ 61.0 | 26 | 8,831 | 73 | .014813 | .002905 |
| < 61.0 | 7 | 11,700 | 104 | .019757 | .007467 |
| At 21 months |  |  |  |  |  |
| ≥ 61.0 | 25 | 9,164 | 77 | .012237 | .002447 |
| < 61.0 | 6 | 11,267 | 100 | .017974 | .007338 |

**Table S2**

*Pearson Correlation And Partial Correlation with and Without Imputation, Paired with the Number of Samples Included*

| Clinical values | N | Pairwise Pearson correlation | N | Partial correlation | N | Pairwise Pearson correlation pooled from 200 multiple imputations | N | Partial correlation pooled from 200 multiple imputations |
| --- | --- | --- | --- | --- | --- | --- | --- | --- |
| HbA_1c_ at onset | 29 | -0.14 (-0.48, 0.24) |  |  | 41 | -0.17 (-0.63, 0.43) |  |  |
| HbA_1c_ at 3 months | 51 | 0.03 (-0.25, 0.30) | 29 | 0.19 (-0.19, 0.52) | 51 | 0.03 (-0.25, 0.30) | 41 | 0.06 (-0.34, 0.45) |
| HbA_1c_ at 6 months | 50 | -0.16 (-0.42, 0.12) | 29 | -0.24 (-0.56, 0.14) | 51 | -0.13 (-0.45, 0.20) | 40 | -0.13 (-0.50, 0.25) |
| HbA_1c_ at 9 months | 50 | -0.22 (-0.47, 0.06) | 28 | -0.44 (-0.70, -0.08) | 50 | -0.22 (-0.47, 0.06) | 40 | -0.22 (-0.55, 0.14) |
| HbA_1c_ at 12 months | 44 | -0.61 (-0.78, -0.37) | 27 | -0.54 (-0.77, -0.19) | 46 | -0.61 (-0.78, -0.36) | 40 | -0.63 (-0.81, -0.33) |
| HbA_1c_ at 15 months | 34 | -0.52 (-0.73, -0.22) | 22 | -0.37 (-0.68, 0.07) | 40 | -0.52 (-0.75, -0.18) | 40 | -0.50 (-0.75, -0.13) |
| HbA_1c_ at 18 months | 33 | -0.56 (-0.76, -0.26) | 22 | -0.45 (-0.73. -0.04) | 40 | -0.54 (-0.77, -0.13) | 40 | -0.51 (-0.77, -0.08) |
| HbA_1c_ at 21 months | 31 | -0.53 (-0.75, -0.23) | 21 | -0.31 (-0.65, 0.14) | 40 | -0.53 (-0.77, -0.18) | 40 | -0.51 (-0.77, -0.11) |

*Note.* The 95% confidence intervals of estimates are given in parentheses.

**Table S3**

*Point Bi-serial Correlations Between the GARF Scores and Clinical Variables*

| Variable | N | Pearson | Sig. (2-tailed) |
| --- | --- | --- | --- |
| GARF average | 51 | 1 |  |
| Reference to mental health services | 51 | .115 | .422 |
| Poor control | 51 | .197 | .166 |
| Episodes of severe hypoglycemia | 51 | -.159 | .265 |
| Insulin resistance | 51 | -.179 | .210 |
| Calls to clinic | 51 | -.263 | .062 |
